# Repeated anodal high-definition transcranial direct current stimulation over the left dorsolateral prefrontal cortex in mild cognitive impairment patients increased regional homogeneity in multiple brain regions

**DOI:** 10.1371/journal.pone.0256100

**Published:** 2021-08-13

**Authors:** Fangmei He, Youjun Li, Chenxi Li, Liming Fan, Tian Liu, Jue Wang

**Affiliations:** 1 The Key Laboratory of Biomedical Information Engineering of Ministry of Education, The Key Laboratory of Neuro-informatics and Rehabilitation Engineering of Ministry of Civil Affairs, and Institute of Health and Rehabilitation Science, School of Life Science and Technology, Xi’an Jiaotong University, Xi’an, Shaanxi, P. R. China; 2 National Engineering Research Center for Healthcare Devices, Guangzhou, Guangdong, P. R. China; BG-Universitatsklinikum Bergmannsheil, Ruhr-Universitat Bochum, GERMANY

## Abstract

Transcranial direct current stimulation (tDCS) can improve cognitive function. However, it is not clear how high-definition tDCS (HD-tDCS) regulates the cognitive function and its neural mechanism, especially in individuals with mild cognitive impairment (MCI). This study aimed to examine whether HD-tDCS can modulate cognitive function in individuals with MCI and to determine whether the potential variety is related to spontaneous brain activity changes recorded by resting-state functional magnetic resonance imaging (rs-fMRI). Forty-three individuals with MCI were randomly assigned to receive either 10 HD-tDCS sessions or 10 sham sessions to the left dorsolateral prefrontal cortex (L-DLPFC). The fractional amplitude of low-frequency fluctuation (fALFF) and the regional homogeneity (ReHo) was computed using rs-fMRI data from all participants. The results showed that the fALFF and ReHo values changed in multiple areas following HD-tDCS. Brain regions with significant decreases in fALFF values include the Insula R, Precuneus R, Thalamus L, and Parietal Sup R, while the Temporal Inf R, Fusiform L, Occipital Sup L, Calcarine R, and Angular R showed significantly increased in their fALFF values. The brain regions with significant increases in ReHo values include the Temporal Inf R, Putamen L, Frontal Mid L, Precentral R, Frontal Sup Medial L, Frontal Sup R, and Precentral L. We found that HD-tDCS can alter the intensity and synchrony of brain activity, and our results indicate that fALFF and ReHo analysis are sensitive indicators for the detection of HD-tDCS during spontaneous brain activity. Interestingly, HD-tDCS increases the ReHo values of multiple brain regions, which may be related to the underlying mechanism of its clinical effects, these may also be related to a potential compensation mechanism involving the mobilization of more regions to complete a function following a functional decline.

## Introduction

Mild cognitive impairment (MCI) refers to the clinical state of a person who suffers from a deficit but otherwise functions normally and does not meet the diagnostic criteria for dementia [1]. MCI is a progressive state between normal aging and Alzheimer’s disease (AD), it is a high-risk state of AD. MCI is often considered a critical time window for predicting early conversion to AD [2]. It has been documented that more than one-tenth of individuals with amnestic MCI have a risk of progressing to AD within two years [3]. Early recognition and intervention of MCI are very important. Thus far, no effective drugs are available for MCI [4]. However, the application of non-invasive brain stimulation techniques such as transcranial direct current stimulation (tDCS) appears to be promising [5].

Non-invasive stimulation techniques include transcranial magnetic stimulation, tDCS, transcranial alternating current stimulation, transcranial pulse stimulation, and transcranial ultrasound stimulation. These techniques have been used by researchers as interventions to ameliorate memory deficits [5]. Compared with transcranial magnetic stimulation, tDCS has been more widely used because of its convenience, better safety, fewer side effects, and low cost. tDCS is thought to alter the resting membrane potential of neurons and regulate neuronal excitability, resulting in an increase or decrease in spontaneous discharge [6]. The anode usually causes an increase in neuronal excitability, whereas the cathode causes a decrease in neuronal excitability [7]. The post-stimulation effect of tDCS lasts for minutes to hours, the effect of repeated stimulation may last from days to months. It has been proposed that repeated stimulation induces neuroplasticity by promoting the remodeling of brain circuits and subsequently improving brain functions including learning and memory [8]. The combination of tDCS and training with specific goals enhances the excitability of task-related brain networks and reconstructs specific neural circuitry [9].

A previous study has shown that anodal-tDCS improved age-related cognitive deficits [10]. Thus, tDCS may also be effective in individuals with MCI by modulating electrophysiological and functional neural activation [11]. Several studies have shown that anodal-tDCS over the left dorsolateral prefrontal cortex (L-DLPFC) could improve working memory, attention, and emotional functions in older adults with amnestic mild cognitive impairment [12–15]. In dementia, a single use of tDCS improves recognition memory [16] and visual recognition memory [17]. These effects were also observed after one month of tDCS application in daily sessions for five days [18]. The effectiveness of tDCS in improving cognitive deficits in disorders such as dementia, depression, and schizophrenia has been evaluated in a variety of original studies and meta-analyses [6, 19–22]. The degree of effectiveness appears to be controversial in different types of dementia [19, 20]. Another study failed to improve apathy and global cognitive function in AD when the L-DLPFC was targeted [21]. Finally, one study assessing the effect of memory training and tDCS concomitantly performed did not reveal any additive impact of cortical stimulation [22]. One explanation for such great variation is the stimulation itself. Conventional tDCS uses large electrodes that are spaced far apart, and both the region of interest and surrounding structures are stimulated. Accordingly, researchers have attempted to apply stimulations with high-definition tDCS (HD-tDCS), which uses compact scalp electrodes to generate a more concentrated current than the conventional tDCS [23]. HD-tDCS is a novel approach that uses arrays of smaller electrodes whose configuration can be optimized for targeting. In particular, a 4×1 ring montage of HD-tDCS has been proposed for unidirectional and targeted stimulation, with the polarity (anode or cathode) set by a center electrode and the area of cortical modulation restricted by adjusting the radii of four return electrodes. The advantages of HD-tDCS compared with conventional tDCS are as follow: First, it is more precisely stimulates a target cortical region to potentially increase the long-term excitability aftereffects [24]. Second, the time course of the respective excitability alterations differed between conventional and HD-tDCS. After conventional tDCS, according to the stimulation polarity, to a larger extent immediately after tDCS and then gradually returned to baseline level. In contrast, plasticity induced by HD-tDCS reached the peak of the respective excitability alteration at about 30 minutes after stimulation. Moreover, the after-effects lasted at least 30 minutes longer than those obtained with conventional tDCS [24, 25]. Third, a more focused approach may allow for tailoring of stimulation to individual indications and symptoms in a way that is not possible with diffuse stimulation [26]. Finally, a more focal intervention might be associated with increased safety. It could potentially reduce the likelihood of side effects due to decreased stimulation of adjacent regions, thus allowing for stimulation with increasing intensity or repetition to enhance efficacy [27]. Kuo et al. have confirmed that when the HD-tDCS 4×1 montage is centered on primary motor cortex, the motor evoked potential amplitude is more profound and lasting than bipolar stimulation [28]. In addition, HD-tDCS introduces less discomfort and improves the applicability for the elderly [29]. In summary, HD-tDCS has broad application prospects. Therefore, in the current study, we used repeated HD-tDCS to explore its impact on spontaneous brain activity of individuals with MCI and the cumulative effect after multiple stimulations.

Neuroimaging technology can identify abnormalities in brain structure and function earlier than functional deficits can be detected [30, 31]. The amplitude of low-frequency fluctuation (ALFF) and regional homogeneity (ReHo) are two reliable algorithms of whole-brain rs-fMRI signals and both a high test-retest reliability. ALFF is defined as the total power within the frequency range between 0.01 and 0.1 Hz and is considered as an effective approach to detect the regional intensity of spontaneous fluctuations and to reflect spontaneous brain activity in the BOLD signal of the rs-fMRI [30]. fractional ALFF (fALFF) measures the relative contribution of low-frequency fluctuations within a specific frequency band to the whole detectable frequency range [32]. It is a standardized and corrected ALFF index that can improve the sensitivity and specificity of detecting spontaneous brain activity by surpassing physiological noise especially in the perivascular, ventricular, and midbrain aqueduct regions [33]. Recently, low-frequency oscillation has attracted extensive attention and has been widely used to explore the resting-state function of the human brain [34]. ReHo uses Kendall’s coefficient of concordance (KCC), measures the similarity of the time series of each voxel and the time series of its neighboring voxels based on the hypothesis that brain activity occurs in clusters. ReHo indicates the degree of synchronized oscillation of neurons in the given brain region [35]. ReHo has been successfully used to study the brain function of patients with mild cognitive impairment and depression [36, 37].

fALFF is used to measure the spontaneous fluctuation amplitude of bold-fMRI signal intensity in a given area of the brain, reflecting the level of spontaneous activity of each voxel in the resting state [32, 38]. ReHo indicates the degree of synchronized oscillation of neurons in the given brain region [39, 40]. The combination of fALFF and ReHo not only reflects the spontaneous fluctuation range of a voxel itself but also reflects the consistency of the time series of a voxel with the surrounding voxels [41, 42]. HD-tDCS will affect the spontaneous activity of some voxels in the resting state and the temporal consistency with surrounding voxels, fALFF and ReHo can detect these resting-state changes [43]. The combination of fALFF and ReHo can better explore the mechanism of tDCS affecting resting brain function. In the current study, we adopted a combined approach.

The current study aimed to explore the effect of repeated anodal HD-tDCS on cognitive function by analyzing the data collected from neuropsychological tests and rs-fMRI data in individuals with MCI. We hypothesized that 1) repeated anodal HD-tDCS causes changes in the resting state of the brain. 2) alterations in spontaneous brain activity are associated with cognitive performance.

## Materials and methods

### Ethical statement

This study was approved by the Medical Ethics Committee of Xijing Hospital Affiliated with the Air Force Military Medical University. All procedures were performed in accordance with the Declaration of Helsinki and registered at ClinicalTrials.gov (ChiCTR2000036603). Participants were included only after informed consent was obtained after explaining the study procedures in detail.

### Participants

We recruited the participants through two channels: outpatients of the Xijing Hospital and other participants through an advertised recruitment program from January 2016 to December 2018, the age of participants ranged from 55 to 85 years. Individuals with MCI were diagnosed based on criteria by Petersen et al. [44] who defined MCI as follows: 1) complaints of memory decline, confirmed by relatives who are familiar with the participant; 2) objective memory impairment; 3) without impairment or with minimum impairments on daily life activity; 4) Mini-mental State Examination (MMSE) score >26 points and Clinical Dementia Rating (CDR) < 0.5; and 5) lack of meeting the criteria for dementia according to the DSM-IV (Diagnostic and Statistical Manual of Mental Disorders, 4th edition, revised). Individuals with MCI were diagnosed independently by two senior neurologists. The following exclusion criteria were used: 1) sudden and stroke-like onset; 2) onset of local nervous system symptoms (hemiplegia, sensory impairment, visual field defect, and abnormal gait) in the early stage of the disease or epilepsy; and 3) dementia and mental illness (such as depression and schizophrenia) caused by systemic diseases. Criteria for termination of participation in the study were based on safety considerations for the participants. If the participant showed unfavorable effects, such as worsening of the disease, serious adverse events, and poor compliance, their participation was terminated.

We initially recruited forty-eight individuals with MCI, the patients were randomly assigned to an anodal HD-tDCS group and a sham group according to a computer-generated randomization list. Three participants were excluded due to not completing the entire trial, and two participants were excluded due to excessive head movements during the scan (head movements > 3 mm or > 3°), the other individuals with MCI were included in the final analysis. None of the participants received any medications that could affect their cognition functions.

### Neuropsychological assessment

All participants underwent a series of neuropsychological tests at baseline and after the stimulation. The series of standardized neuropsychological tests included the MMSE, Montreal Cognitive Assessment (MoCA). All clinical evaluations were double-blinded.

### HD-tDCS

A battery-powered constant-current direct current stimulator (1300 A and 4 × 1-C3A, Soterix Medical, New York, NY, USA) was used to deliver 1mA HD-tDCS in each intervention. Participants in the HD-tDCS group received HD-tDCS in the L-DLPFC. To stimulate the L-DLPFC, the anode electrode was placed over F3 according to the 10–20 international system for EEG electrode placement, surrounded by four cathodes at a radius of 3 cm, with high-definition mini electrodes [14, 19], with a constant current of 1.0 mA intensity lasting for 20 minutes. Stimulation was performed 5 times a week for 2 weeks during the daytime. The same montage was used for the sham condition. Sham group was applied at 1mA for 1 minute, including 30 seconds in the beginning for ramping up to 1mA and then the current ramping down to 0mA in 30 seconds, at the last 1 minute, the current ramping up to 1 mA in 30 seconds and then ramping down again to 0mA at the end of the sham period for the last 30 seconds. Sham stimulation let the participants produce the same current change as in the HD-tDCS group. The participants were not aware of these changes because the conditions were automatically programmed and automatically executed. Adverse effects of HD-tDCS were assessed at the end of each session using a structured HD-tDCS adverse effects questionnaire [15]. The adverse effects in the questionnaire included headache, scalp pain, tingling, itching, burning, skin redness, trouble concentrating, skin lesions, and sleepiness. Individuals with MCI, HD-tDCS operators, and clinical raters were kept blind to treatment conditions until this unblinding; patients were not asked to guess the condition, and raters were not involved in HD-tDCS application.

### Image acquisition

All participants were imaged with a 3-Tesla MRI system (750 General Electric Medical Systems, Milwaukee, WI, USA) at the Department of Radiology of Xijing Hospital, Air Force Medical University, Xi’an, China. Before imaging, participants were asked to keep their eyes closed and relaxed, stay awake and move as little as possible during the imaging. A standard birdcage head coil was used, and restraint foam pads were used to minimize head movement and reduce scanner noise. The resting-state functional images were obtained using a gradient-echo planar imaging sequence with the following parameters: repetition time (TR) = 2000ms, echo time (TE) = 30ms, image matrix = 64×64, slice thickness = 3.5mm, flip angle (FA) = 90°, field of view (FOV) = 240mm×240mm, layers = 45. Three-dimensional (3D) anatomical data were acquired using a 3D Bravo T1-weighted sequence equipped with an eight-channel receiver head coil, with the following parameters: repetition time = 8.2 ms, echo time = 3.2 ms, field of view (FOV) = 240 mm×240 mm, slice thickness = 1 mm, resolution = 256×256×256.

### rs-fMRI data preprocessing

Data preprocessing was conducted using the Data Processing Assistant for Resting-State fMRI (DPARSF) toolbox [45], which based on Statistical Parametric Mapping (SPM12, Well come Department of Cognitive Neurology, London, UK) [46] implemented in MATLAB (R2013a, The Math Works, Natick, MA). The preprocessing steps were as follows: 1) first, the first 10 time points were removed; 2) slice timing; 3) head motion correction. In this study, participants with head movements > 3 mm or > 3° between the two volumes were excluded; 4) spatial normalization: the standard EPI template was used for spatial normalization (3 × 3 × 3 mm); 5) spatial smoothing was conducted by applying a 6 mm full-width half-maximum Gaussian filter. Head motion parameters, white matter signal, and cerebrospinal fluid signal were included to regress out nuisance covariates; 6) the linear drift was also removed.

### Calculation of fALFF

The fALFF provides information on the magnitude of brain activity of each brain region within a network of interest. It represents the ratio of the fluctuation of the BOLD signal in the low-frequency range to the entire frequency range [32]. Calculation routines are available from the DPARSF toolbox [45]. To calculate the fALFF, the standard procedure provided by Zou was followed [32]. First, the functional images were spatially smoothed with a Gaussian kernel of 6 mm full width at half maximum. Second, the time series of each voxel was converted to the frequency domain to obtain the power spectrum. The ALFF within the range (0.01–0.1 Hz) was then calculated as the square root of the power spectrum [47]. Finally, the ratio between the ALFF and the power spectrum of the entire frequency range was calculated to obtain the fALFF measurements. For standardization, a single fALFF graph was subtracted from its average value and divided by the standard deviation to convert it into a z-score.

### Calculation of ReHo

ReHo analysis was performed using the DPARSF toolbox [45]. According to the hypothesis that intrinsic brain activity occurs in a mass or region made up of many cluster volumes, Zang et al. [47] proposed ReHo. We used Kendall’s coefficient of concordance (KCC) to measure regional homogeneity or similarity of the ranked time series of a given voxel with its nearest 26 neighbor voxels in a voxel-wise way:

$$W=\frac{\sum {\left(R_{i}\right)}^{2}-n{\left(\bar{R}\right)}^{2}}{\frac{1}{12}K^{2}(n^{3}-n)}$$

where W is the KCC among given voxels, ranging from 0 to 1; Ri is the sum rank of the “i”th time point; $\bar{R}$ = ((n + 1) K)/2 is the mean of the Ris; K is the number of time series within a measured cluster (27, one given voxel plus the number of its neighbors); and n is the number of ranks. KCC-ReHo has a value between 0 and 1. Higher values indicate better local synchronization. Then, the resulting ReHo map was normalized by the mean ReHo value within the brain mask. For standardization, a single ReHo graph was subtracted from its average value and divided by the standard deviation to convert it into a z-score.

### Statistical analysis

Statistical analysis was performed using SPSS software version 16.0 (statistical program for social sciences, SPSS Inc. Chicago, IL) for demographic and clinical data, and fMRI data’s statistical analysis was performed using SPM12 (http://www.fil.ion.ucl.ac.uk/spm).

For fMRI data, a two-sample *t*-test was performed to examine the voxel-wise difference between the HD-tDCS and sham groups using the Resting-State fMRI Data Analysis (REST) toolbox [48]. (The statistical threshold was set at the voxel level with P<0.05, for multiple comparisons using gaussian random field theory voxels P<0.01 and cluster size of >54 voxels, AlphaSim corrected). In addition, we used a paired *t*-test to explore the differences in each group before and after the sessions. These voxels were regarded as regions of interest, showing significant differences between the two groups. The intervention effect of HD-tDCS was calculated by conducting a repeated-measures analysis of variance (ANOVA) on the neuropsychological test scores. Condition (HD-tDCS vs. sham stimulation) was taken up as a between-subject factor and time (before vs. after sessions) as a within-subject factor. After statistical results were obtained, a multiple comparison correction was performed with Gaussian random field correction (GRF) (voxel-level setting of P<0.01, and cluster-level setting of P <0.05). The specific anatomical positions of the brain regions with statistical significance on the corresponding MNI coordinates were determined using the viewer tool within the REST software. The voxels of different brain regions were obtained, and the results were presented using REST software.

Finally, regions showing significant differences were extracted as regions of interest (ROIs). ReHo and fALFF values were subsequently extracted from these seed regions within each participant, and Pearson correlations were calculated to measure the association between the group mean ReHo and fALFF values within ROIs and MoCA, as well as MMSE scores at P< 0.05, Bonferroni corrected.

## Results

### Demographic and clinical data

The groups did not differ in gender, age, years of education, MMSE, and MoCA scores. Forty-three participants were included in the final analysis, including twenty-four in the HD-tDCS group and nineteen in the sham group. The demographic characteristics of the participants are presented in Table 1.

**Table 1:** Demographic and neuropsychological data.

	MCI HD-tDCS	MCI sham stimulation	P*
Sample size(n)	24	19	
Age (mean ± SD)	63.5 ±4.80	65.63 ±3.53	0.113^b^
Gender (Males/Females)	24(7/17)	19(4/15)	0.728^a^
Year of education (years)	10.38±3.06	9±2.45	0.119^b^
MMSE score (Mean ± SD)	25.13 ±0.79	24.89±1.1	0.431^b^
MoCA scores (Mean ± SD)	22.13±1.36	22.05±1.65	0.875^b^

Data are expressed as mean ± standard error (SE). No significant differences (P>0.05) were observed between the HD-tDCS group and sham group in age, gender, years of education, MMSE, and MoCA.

a: The P-value of gender was obtained using the chi-square test.

b: The P-value ware obtained using a two-sample t-test. HD-tDCS, high-definition transcranial direct current stimulation; MMSE, The Mini-mental State Examination; MoCA, Montreal Cognitive Assessment; MCI, Mild cognitive impairment. All statistical analyses were conducted using SPSS software version 16.0 (statistical program for social sciences, SPSS Inc. Chicago, IL).

There is no significant difference before and after HD-tDCS stimulation in the MMSE scores and the MoCA scores. Similarly, there is no significant difference before and after sham stimulation in the MMSE scores and the MoCA scores. The results are shown in Table 2.

**Table 2:** The results of the neuropsychological assessment pre-post intervention in both MCI groups.

	Pre-HD-tDCS	Post-HD-tDCS	P*	Pre-sham	Post-sham	P*
MMSE score (Mean ± SD)	25.13 ±0.79	25.21±0.81	0.083^c^	24.89±1.10	25.05±1.01	0.163^c^
MoCA scores (Mean ± SD)	22.13±1.36	22.17±1.38	0.328^c^	22.05±1.65	22.19±1.39	0.103^c^

Data are expressed as mean ± standard error (SE), No significant difference (P>0.05) was found before and after HD-tDCS intervention. Similarly, no significant difference (P>0.05) was found before and after the sham intervention.

c: The P-value ware obtained using Paired t-test. MCI, Mild cognitive impairment; HD-tDCS, high-definition transcranial direct current stimulation; MMSE, The Mini-mental State Examination; MoCA, Montreal Cognitive Assessment; All statistical analyses were conducted using SPSS software version 16.0 (statistical program for social sciences, SPSS Inc. Chicago, IL).

### fALFF analysis

**Fig 1:**
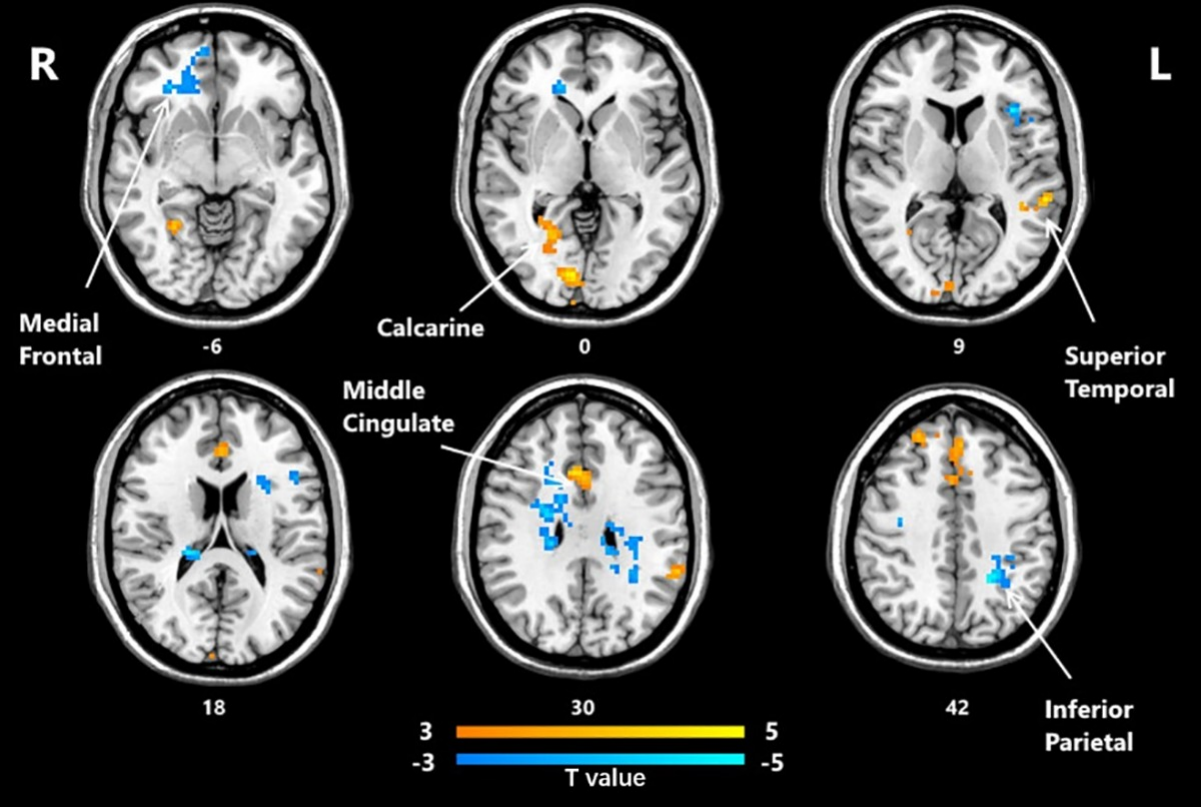
Brain regions that showed significant alterations in fALFF between the group post- HD-tDCS and post-sham group (P< 0.05, AlphaSim corrected). The yellow color denotes HD-tDCS> sham-HD-tDCS in the fALFF values and the blue color denotes HD-tDCS < sham-HD-tDCS. The T-value bars are shown at the bottom. The brain regions with significant increases in fALFF values include Calcarine R, Temporal Sup L, Cingulum Mid R, Frontal Sup Medial R. However, Frontal Med Orb R, Frontal Inf Tri L, Precentral R, Parietal Inf L, and Frontal Inf Oper R decreased significantly. fALFF, fractional amplitude of low-frequency fluctuation; HD-tDCS, high-definition transcranial direct current stimulation.

**Fig 2:**
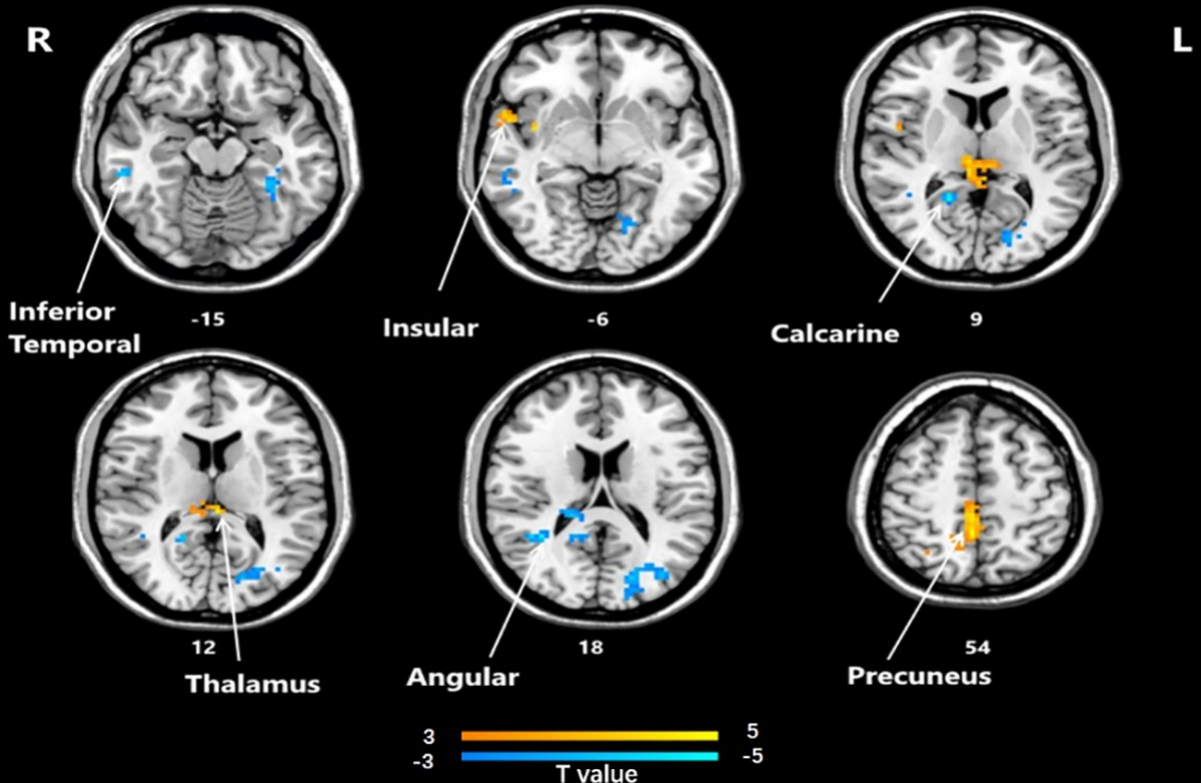
Brain regions that showed significant alterations in fALFF before and after HD-tDCS. (P<0.05, AlphaSim corrected). The yellow color denotes post-HD-tDCS> pre-HD-tDCS in the fALFF values and the blue color denotes post-HD-tDCS < pre-HD-tDCS. The T-value bars are shown at the bottom. The brain regions with significant increases in fALFF include Temporal Inf R, Fusiform L, Occipital Sup L, Calcarine R, Angular R. However, Insula R, Precuneus R, Thalamus L, and Parietal Sup R decreased significantly. fALFF, fractional amplitude of low-frequency fluctuation; HD-tDCS, high-definition transcranial direct current stimulation.

**Fig 3:**
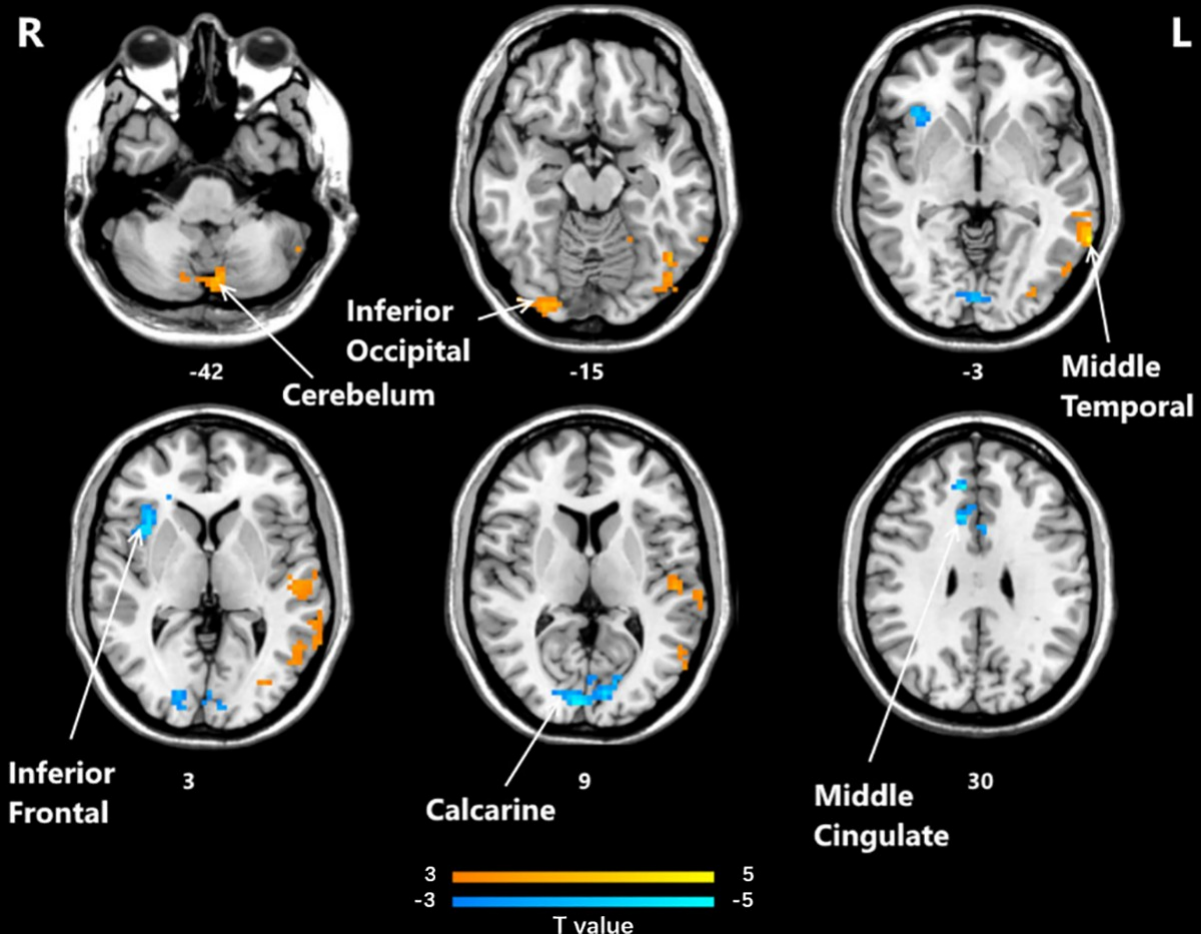
Brain regions that showed significant alterations in fALFF values before and after sham stimulation. (P<0.05, AlphaSim corrected). The yellow color denotes post-sham-HD-tDCS> pre-sham-HD-tDCS in the fALFF values and the blue color denotes post-sham-HD-tDCS < pre-sham-HD-tDCS. The T-value bars are shown at the bottom. The brain regions with significant increases in fALFF include Frontal Inf Oper R, Calcarine R, Cingulum Mid R. However, Cerebellum 7b L, Temporal Mid L, Cerebellum Crus1 L, Occipital Inf R, and Postcentral L decreased significantly. fALFF, fractional amplitude of low-frequency fluctuation; HD-tDCS, high-definition transcranial direct current stimulation.

The significant differences in fALFF between the post-HD-tDCS group and post-sham stimulation group, pre-HD-tDCS group and post-HD-tDCS group, pre-sham group, and post-sham group are summarized in Table 3 and Figs 1–3. The changes of fALFF in individuals with MCI post-HD-tDCS group and the post-sham group were compared as shown in Table 3 and Fig 1 (P< 0.05, AlphaSim corrected). The brain regions with significant increases in fALFF included the Calcarine R, Temporal Sup L, Cingulum Mid R, and Frontal Sup Medial R regions. However, the Frontal Med Orb R, Frontal Inf Tri L, Precentral R, Parietal Inf L, and Frontal Inf Oper R regions fALFF significantly decreased. The changes of fALFF in individuals with MCI before and after HD-tDCS were compared as shown in Table 3 and Fig 2 (P< 0.05, AlphaSim corrected). We found the brain regions with significant increases in fALFF include the Temporal Inf R, Fusiform L, Occipital Sup L, Calcarine R, and Angular R regions. However, the fALFF values of the Insula R, Precuneus R, Thalamus L, and Parietal Sup R decreased significantly. The changes of fALFF in individuals with MCI before and after sham stimulation were compared as shown in Table 3 and Fig 3 (P< 0.05, AlphaSim corrected), The brain regions with significant increases in fALFF included the Frontal Inf Oper R, Calcarine R, Cingulum Mid R regions. However, in areas such as the Cerebellum 7b L, Temporal Mid L, Cerebellum Crus1 L, Occipital Inf R, and Postcentral L regions, there were decreased significantly.

**Table 3:** Regions with fALFF differences between each two groups.

Brain areas	MNI coordinates			fALFF		
	X	Y	Z	BA	Peak voxels	Tvalue
Post-HD-tDCS versus post-sham						
post-HD-tDCS > post-sham						
Calcarine R	9	-84	0	18	157	4.35
Temporal Sup L	-57	-36	9	40	70	4.07
Cingulum Mid R	6	21	30	32	204	3.95
Frontal Sup Medial R	12	45	45	8	76	3.19
post-HD-tDCS < post-sham						
Frontal Med Orb R	30	36	-6	10	73	-3.47
Frontal Inf Tri L	-36	21	9	13	73	-4.44
Precentral R	21	-30	18	6	72	-4.17
Parietal Inf L	-21	-48	42	40	210	-4.29
Frontal Inf Oper R	27	0	27	32	92	-4.06
Pre-HD-tDCS versus post-HD-tDCS						
Pre-HD-tDCS > post-HD-tDCS						
Insula R	39	0	-6	22	56	3.41
Precuneus R	3	-45	54	7	155	3.88
Thalamus L	-3	-30	12		62	3.71
Parietal Sup R	36	-51	60	7	66	3.52
Pre-HD-tDCS < post-HD-tDCS						
Temporal Inf R	57	-33	-15	20	56	-3.64
Fusiform L	-24	-69	-9	36	60	-3.93
Occipital Sup L	-18	-81	21	19	239	-4.69
Calcarine R	18	-51	9		65	-4.51
Angular R	36	-48	18		54	-4.56
Pre-sham versus post-sham						
Pre-sham > post-sham						
Cerebellum 7b L	-6	-75	-42		85	4.63
Temporal Mid L	-66	-51	-3	21	369	7.11
Cerebellum Crus1 L	-9	-69	-30		125	4.83
Occipital Inf R	30	-90	-15	18	83	4.28
Postcentral L	-36	-33	42	7	254	4.83
Pre-sham < post-sham						
Frontal Inf Oper R	39	15	3	13	70	-3.75
Calcarine R	6	-90	9	18	138	-4.18
Cingulum Mid R	9	39	30	9	224	-4.36

fALFF, fractional amplitude of low-frequency fluctuation; HD-tDCS, high-definition transcranial direct current stimulation; MNI, Montreal neurological institute; BA, Brodmann area. P< 0.05, Alphasim corrected.

### Interaction

**Fig 4:**
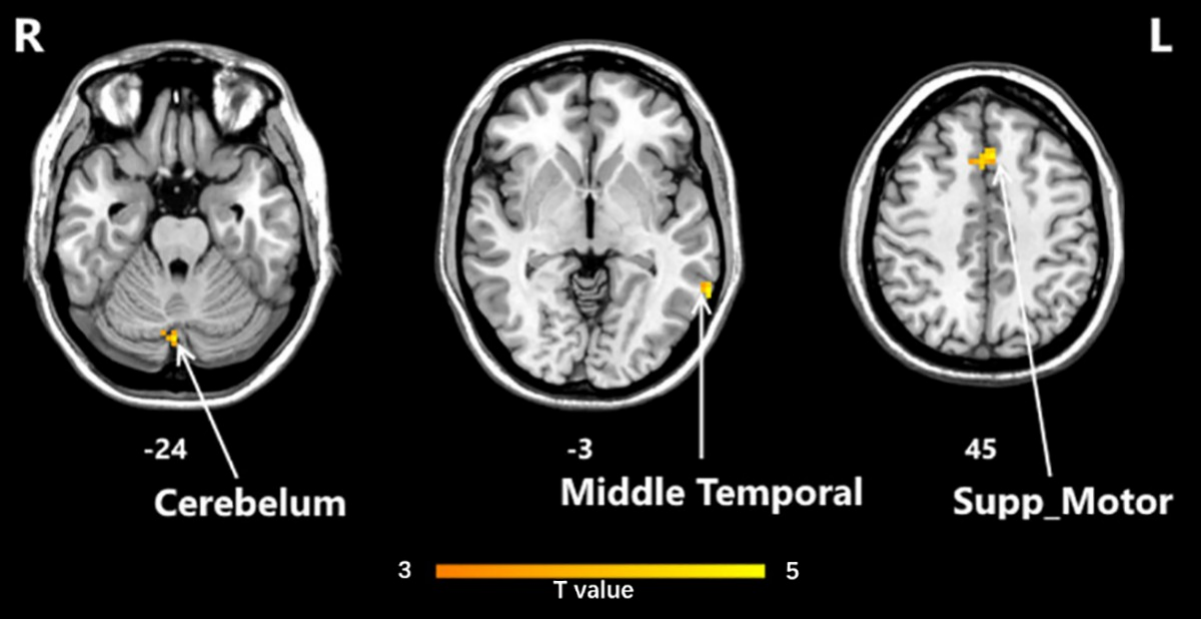
fALFF interaction area as the picture shows were cerebellum, Middle Temporal, and sup-motor (P<0.05, AlphaSim corrected). fALFF, the fractional amplitude of low-frequency fluctuation.

Using mixed effect analysis, it was found that the HD-tDCS group and sham groups had opposite directions for fALFF value regulation in the Middle Temporal Gyrus, Cerebellum, and Supp Motor Area positions, as shown in Fig 4.

### ReHo analysis

**Fig 5:**
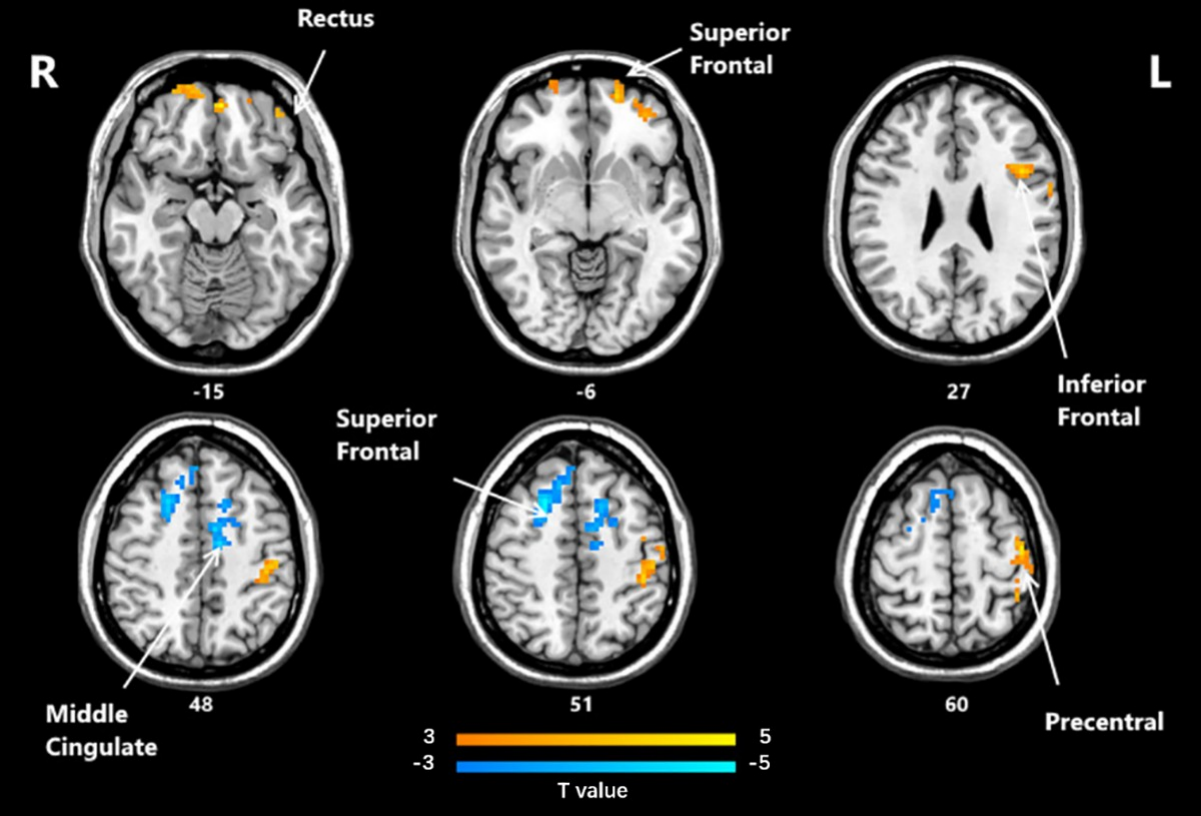
Brain regions that showed significant alterations in ReHo values between the two groups post-HD-tDCS and post-sham stimulation (P< 0.05, AlphaSim corrected). The yellow color denotes HD-tDCS> sham stimulation in the ReHo values and the blue color denotes HD-tDCS < sham stimulation. The T-value bars are shown at the bottom. Compared with HD-tDCS and sham stimulation, the areas with high ReHo values include Rectus L, Frontal Sup Orb L, Precentral L, Frontal Inf Oper L, areas with low ReHo values include Frontal Sup R and Cingulum Mid L. ReHo, regional homogeneity; HD-tDCS, high-definition transcranial direct current stimulation.

**Fig 6:**
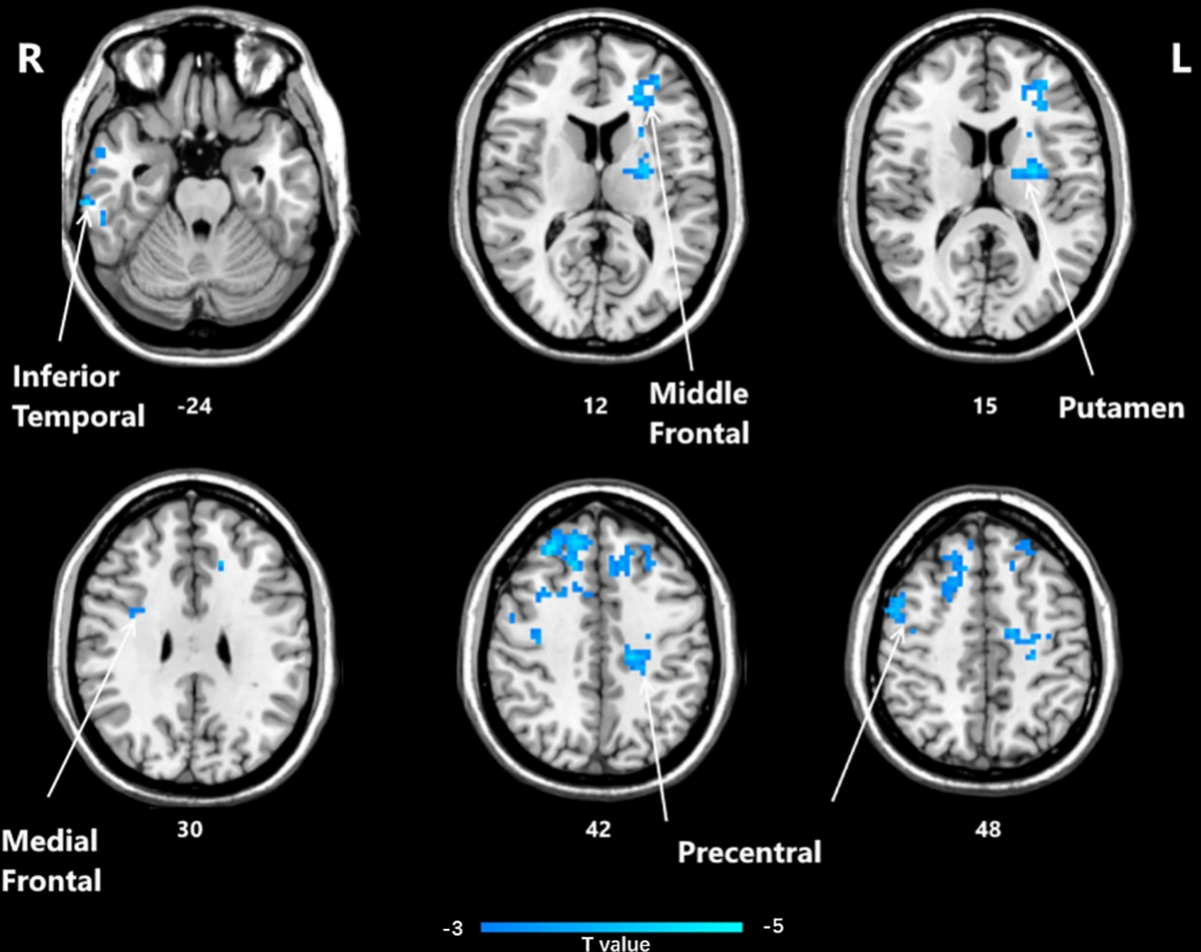
Brain regions that showed significant alterations in ReHo values before and after HD-tDCS. (P<0.05, AlphaSim corrected). The yellow color denotes post-HD-tDCS> pre-HD-tDCS in the ReHo values and the blue color denotes post-HD-tDCS < pre-HD-tDCS. The T-value bars are shown at the bottom. The brain regions with a significant decrease in ReHo values include Temporal Inf R, Putamen L, Frontal Mid L, Precentral R, Frontal Sup Medial L, Frontal Sup R, and Precentral L. ReHo, regional homogeneity; HD-tDCS, high-definition transcranial direct current stimulation.

**Fig 7:**
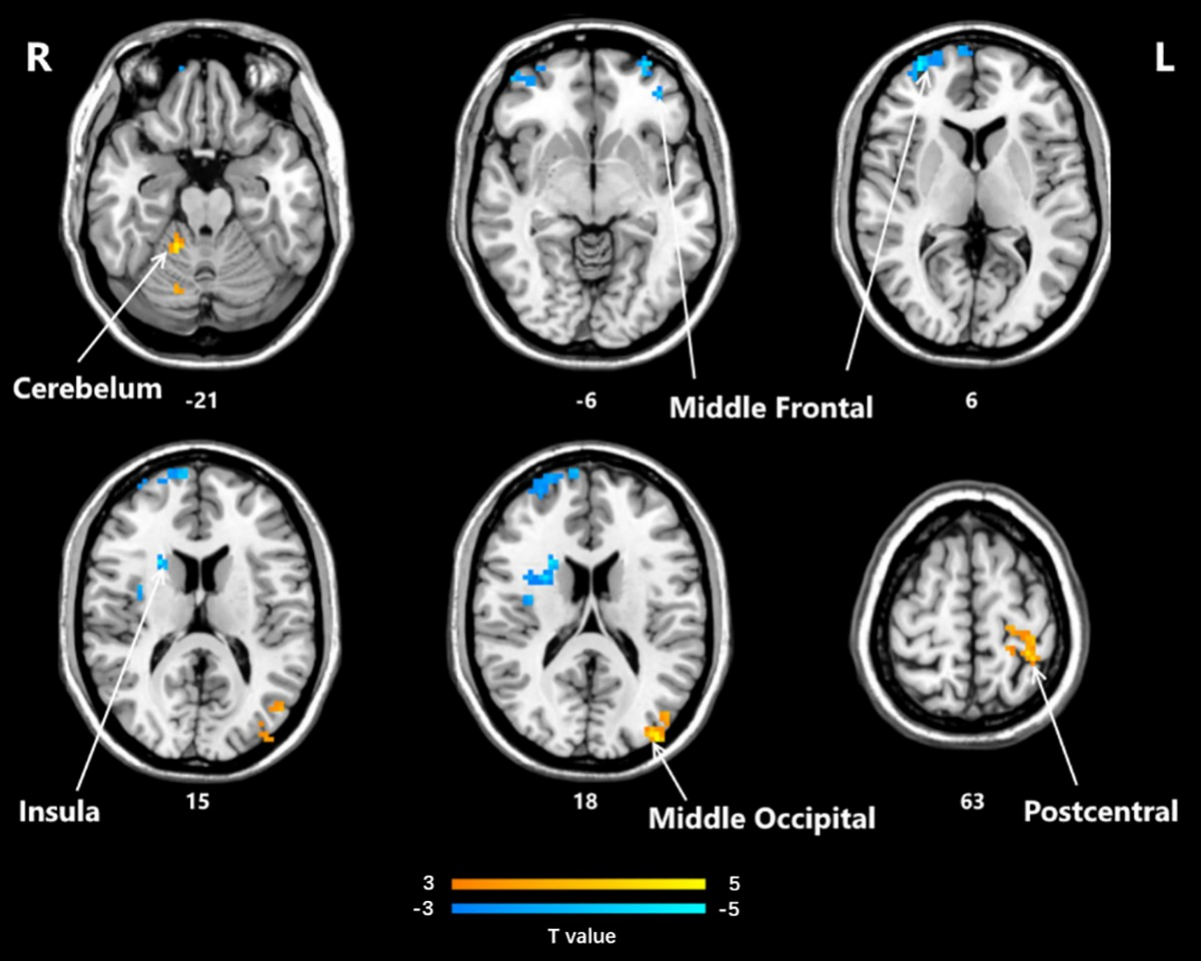
Brain regions that showed significant alterations in ReHo values before and after sham stimulation (P<0.05, AlphaSim corrected). The yellow color denotes post-sham-HD-tDCS> pre-sham-HD-tDCS in the ReHo values and the blue color denotes post-sham stimulation < pre-sham stimulation. The T-value bars are shown at the bottom. The brain regions with significant increases in ReHo values include Frontal Mid R, Frontal Mid Orb L, Insula R. However, Cerebellum 4 5 R, Occipital Mid L, and Postcentral L decreased significantly. ReHo, regional homogeneity; HD-tDCS, high-definition transcranial direct current stimulation.

Significant differences in ReHo between the post-HD-tDCS group and post-sham group, pre-HD-tDCS group and post-HD-tDCS group, pre-sham group, and post-sham group are summarized in Table 4 and Figs 5–7. The changes in ReHo in individuals with MCI post-HD-tDCS group and post-sham group are compared as shown in Table 4 and Fig 5 (P< 0.05, AlphaSim corrected). The brain regions with significant increases in ReHo include Rectus L, Frontal Sup Orb L, Precentral L, Frontal Inf Oper L. However, the areas that decreased significantly in ReHo value include the Frontal Sup R and Cingulum Mid L regions. The changes of ReHo in individuals with MCI before and after HD-tDCS are compared as shown in Table 4 and Fig 6. The brain regions with significant increases in ReHo include Temporal Inf R, Putamen L, Frontal Mid L, Precentral R, Frontal Sup Medial L, Frontal Sup R, and Precentral L regions. The changes of ReHo in individuals with MCI before and after sham stimulation are compared as shown in Table 4 and Fig 7. The brain regions with significant increases in ReHo values include Frontal Mid R, Frontal Mid Orb L, Insula R regions. However, Cerebellum 4 5 R, Occipital Mid L, and Postcentral L regions decreased significantly.

**Table 4:** Regions with ReHo differences between each two groups.

Brain areas	MNI coordinates			ReHo		
	X	Y	Z	BA	Peak voxels	Tvalue
Post-HD-tDCS versus post-sham						
Post-HD-tDCS > post-sham						
Rectus L	-3	54	-15	11	72	4.07
Frontal Sup Orb L	-18	60	-6	11	79	3.41
Precentral L	-45	-12	60	3	177	3.68
Frontal Inf Oper L	-42	12	27	9	62	3.56
Post-HD-tDCS < post-sham						
Frontal Sup R	21	18	51	8	167	-5.24
Cingulum Mid L	-12	-9	48	24	108	-4.38
Pre-HD-tDCS versus post-HD-tDCS						
Pre-HD-tDCS < post-HD-tDCS						
Temporal Inf R	66	-24	-24	21	126	-4.13
Putamen L	-27	-6	15	13	137	-4.32
Frontal Mid L	-24	36	12	10	75	-4.16
Precentral R	54	3	48	6	83	-3.69
Frontal Sup Medial L	-15	30	30	8	98	-3.45
Frontal Sup R	15	45	39	8	199	-5.02
Precentral L	-18	-24	42	31	70	-4.23
Pre-sham versus post-sham						
Pre-sham > post-sham						
Cerebellum 4 5 R	18	-45	-21		126	4.03
Occipital Mid L	-39	-90	18	19	58	4.27
Postcentral L	-33	-39	63	3	64	3.7
Pre-sham < post-sham						
Frontal Mid R	33	63	6	10	196	-4.53
Frontal Mid Orb L	-30	63	-6	10	72	-3.87

ReHo, regional homogeneity; HD-tDCS, high-definition transcranial direct current stimulation; MNI, Montreal neurological institute; BA, Brodmann area. P< 0.05, Alphasim corrected.

### Interaction

**Fig 8:**
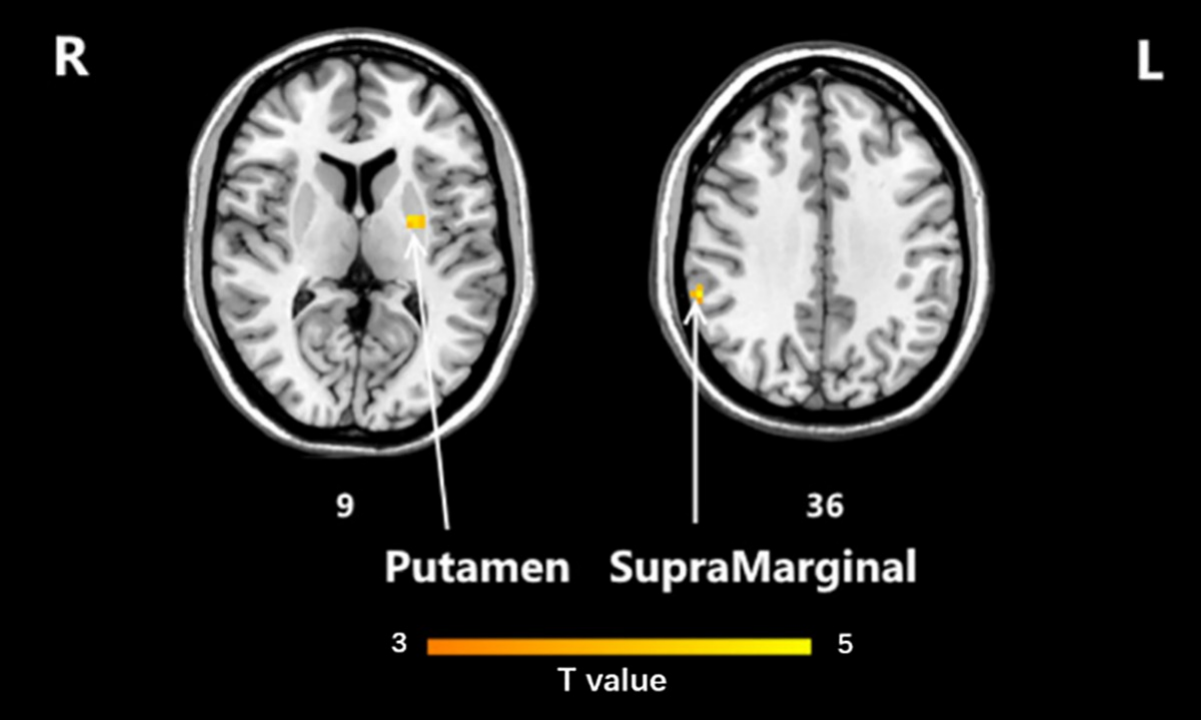
ReHo interaction area as the picture shows was putamen and supramarginal. ReHo, regional homogeneity.

In putamen and supramarginal, the HD-tDCS group and sham groups had opposite directions for ReHo value regulation, as shown in Fig 8.

In summary, in the present study, we found a unique pattern of brain activity alterations in individuals with MCI. The fALFF values of HD-tDCS participants showed decreased in the Insula R, Precuneus R, Parietal sup R, and Thalamus L areas, whereas these values increased in the Fusiform L, Occipital Sup L, Calcarine R, and Angular R regions. Interestingly, after repeated HD-tDCS, the ReHo values increased in multiple areas, such as the Putamen L, Frontal Mid L, Precentral R, Frontal Sup Medial L, Frontal Sup R, and Precentral regions.

### Correlation analysis of clinical scale scores and fALFF values or ReHo values

No significant correlations were found between fALFF or ReHo values and MMSE or MoCA scores between the two groups.

## Discussion

In this study, we examined the changes of fALFF and ReHo in individuals with MCI after HD-tDCS or sham stimulation. We found that several brain regions showed significant differences in terms of their fALFF and ReHo values. We focused on the effect of HD-tDCS, so we paid special attention to the changes before and after HD-tDCS. Compared with the pre-HD-tDCS group, the post-HD-tDCS group had increased fALFF and ReHo values in the Temporal Inf region. Decreased fALFF values in the Insula R, Precuneus R, Parietal Sup R, and Thalamus L region, as well as increased fALFF in the Fusiform L, Occipital Sup L, Calcarine R, and Angular R regions were observed. Interestingly, after repeated HD-tDCS, the ReHo values increased in multiple areas such as the Putamen L, Frontal Mid L, Precentral R, Frontal Sup Medial L, Frontal Sup R, and Precentral.

Using fALFF and ReHo measures, this study explored the effect of repeated HD-tDCS on individuals with MCI. These two methods are based on different neurophysiological mechanisms, with ALFF demonstrating neural intensity and ReHo demonstrating the coherence of neural activity regardless of intensity. fALFF has been used as an alternative indicator of the intensity of spontaneous nerve activity and as a biomarker for neurological disorders. The ReHo analysis showed the consistency of the time series of activities in different regions, fALFF and ReHo are complementary to each other in depicting regional spontaneous neural activity and provide different perspectives in understanding the impact of HD-tDCS on individuals with MCI.

The area where both fALFF and ReHo values increased after HD-tDCS were located in the Temporal Inf R. The coexisting change in functional intensity and activity coherence in the region might represent more obvious functional changes in brain areas than those reflected by only one of these two methods [37]. The increased spontaneous brain activity in the Temporal Inf of individuals with MCI was partially consistent with previous findings [49]. The Temporal Inferior gyrus plays an important role in cognitive learning behavior and memory. The fact that both the fALFF and ReHo values were altered in the temporal inferior gyrus region indicates that this area is the key node in the process of transforming from the default mode network to the task network [50].

### The impact of tDCS on fALFF

Decreased fALFF values were observed in Parietal Sup R, Precuneus R, Insula R, and Thalamus L after HD-tDCS. The Parietal Sup R and Precuneus R make up a component of the default mode network (DMN). Decreased fALFF values in these regions indicated that HD-tDCS allow brain activity that is resting to transition into the task state more easily [51]. One study previously found a close relationship between fALFF and specific behavioral parameters [52]. Keeser et al. found that anodal-tDCS of the DLPFC and cathodal tDCS applied to the right supraorbital area caused significant resting-state network changes, covering areas close to and far from the stimulation electrode [53]. Therefore, their study concluded that HD-tDCS affects brain activity in multiple areas, not just in the cortical area underlying the anode. The precuneus is a very important node of the DMN, and the information from the DMN subsystem is considered to be gathered here [54]. The precuneus plays an important role in ongoing cognitive processes during the resting state and in cognitive activities [55].

The Insula R is a very important node of the salience network (SAN), the increases in fALFF seen in this region will result in negative interpretations of external events. HD-tDCS causes the fALFF value of the Insula R to decrease, which may reduce the negative interpretations of events. This, in turn, helps improve the symptoms of depression in individuals with MCI [56]. The Insula R is also a key node of the right frontoparietal network (RFPN), and the frontoparietal network (FPN) is involved in executive functions, such as attention allocation, working memory, performance monitoring, and planning [57]. HD-tDCS reduces the fALFF value of the left thalamus, which is related to a variety of emotions and cognitive functions. In a rewarding environment, the thalamus mediates reward-based decision-making, emotional drive, planning, and monitoring of target behavioral decisions [58]. The thalamus is also an indispensable part of the cortex-limbic system; the thalamus transmits sensory saliency information to the prefrontal cortex [59]. Therefore, the regulation of HD-tDCS on the thalamus may affect the function execution of different networks.

Increased fALFF values were observed in Occipital Sup L, Fusiform L, Temporal Inf R, Calcarine R, and Angular R regions following HD-tDCS. Increased fALFF of Occipital Sup L and Fusiform L regions is more conducive to recognize external visual information, especially facial recognition abilities, considering that the fusiform gyrus is related to facial recognition abilities. Studies have shown that the activation of the fusiform gyrus is higher when attractive faces were seen [60, 61]. It is considered that a beautiful appearance evokes a wide range of neural networks. This including perception, reward, decision-making, and other circuits [62]. In contrast, when people with autism see a human face, there is little or no excitement in the fusiform gyrus. The Calcarine R, Angular R, and Temporal Inf R regions are part of the inferior longitudinal fasciculus (ILF), and the ILF connects the occipital and temporal lobes, which participate in instant visual memory. The main roles of the ILF include facial recognition, reading, regulation of vocabulary, semantic processes, and emotion regulation. The destruction of ILF integrity is related to cognitive impairment, color discrimination defects, mood disorders, and tremor-based motor symptoms [63]. The increased fALFF values observed in the ILF region after HD-tDCS is likely beneficial for integrating external visual information and completing cognitive tasks.

The influence of HD-tDCS on brain areas is particularly prominent in the three parts of the Middle Temporal Gyrus, Cerebellum, and Supp Motor Area. In the Middle Temporal Gyrus, HD-tDCS activated the spontaneous activity of the local brain. We speculate that HD-tDCS can help patients transition from the default mode network in the resting state to the task-executing network. In the Cerebellum, HD-tDCS activates the spontaneous activity of the brain, and activation of the cerebellum is related to the cognitive circuit, which can help participants to complete tasks well [64, 65]. The Cerebellum is anatomically connected to multiple areas of the frontal cortex and marginal area, which is essential for its participation in cognitive processing [66, 67]. Changes in the structure of the cerebellum can also cause changes in fALFF values. In the Supp Motor area, HD-tDCS increased the fALFF values. The activation of this area was related to the movement plan. The movements produced and controlled by the body are particularly helpful in producing sequential movements in memory. In short, after performing a mixed effect analysis, we found that the Middle Temporal Gyrus, Cerebellum, and Supp Motor Area are key areas for HD-tDCS to regulate cognition function.

### The impact of tDCS on ReHo

Surprisingly, in this study, ReHo values significantly increased after HD-tDCS in multiple regions. We found increased ReHo values in the Temporal Inf R, Precentral R, Frontal Sup R, Precentral L, Putamen L, Frontal Mid L, and Frontal Sup Medial L regions.

The ReHo value reflects the level of functional consistency between a given voxel and its nearest neighbors. It can be used to assess brain activity in a static state and to reveal the complexity of human brain functions. Both the reductions and increases in ReHo values represent abnormal changes in brain activity and indicate different phenomena. Decreased ReHo values indicate that the cerebral blood flow in some areas is reduced, and the change in regional metabolic rate is reduced, as shown in previous studies in individuals with MCI [68–70]. Increased in ReHo values imply a compensation mechanism that is also consistent with the hypothesis of previous studies, which suggested that AD patients can use other neurological resources to compensate for the loss of cognitive function in the early stage of the disease [71]. Regarding the association between ReHo and cognitive integrity, one of the most consistent findings is that the ReHo values of the precuneus are reduced with the progression of AD [72]. Regarding the relationship between ReHo and cognitive function, although many previous findings are contradictory, most people still tend to believe that the better the cognitive function, the larger the ReHo value in the area that plays a key role in the relevant cognitive function. Therefore, the collaborative work of areas that play a key role in cognition helps to better complete cognitive functions. In this study, HD-tDCS up-regulated the ReHo value of multiple brain regions, all of which have been proposed as compensatory reallocation or recruitment region of cognitive resources.

Kang et al. found that individuals with MCI had higher ReHo values in the Bilateral Putamen Region, Posterior Central Gyrus, and left Middle Cingulate Gyrus compared to healthy controls [73]. Increased ReHo in these areas has a compensatory effect on damage to the Medial Temporal Lobe area and marginal structures [74]. The results of this study were partly in accordance with those of Kang. However, we found increased ReHo values in additional areas. For instance, changes in the right dorsolateral superior frontal gyrus are associated with negative emotions [64]. The increased ReHo values in these areas help to perceive external information and participate in completing tasks. We found that the changes in ReHo values of the Putamen and Supra Marginal Gyrus were contrary to those seen during the sham stimulation. At the same time, the Putamen and other basal ganglia structures participate in motor functions, including exercise selection, preparation, and execution [75, 76]. In addition, the putamen and other basal ganglia regions have been shown to play important roles in cognitive and emotional processing [77, 78]. Temporal Inf R, Precentral R, Frontal Sup R, Precentral L, Frontal Mid L, and Frontal Sup Medial L regions are involved in memory and cognitive tasks, and increased ReHo values in these regions may aid in completing more advanced cognitive tasks.

HD-tDCS has the potential to modulate the neurophysiological components of impulsivity [65]. A study indicates that tDCS can enhance processing speed, working memory, and executive functions in patients with mood and schizophrenia-spectrum disorders [79]. However, evidence of a positive effect on general cognitive functioning and memory is either inconclusive in AD or weak in MCI [80, 81]. The results of these studies are sometimes conflicting.

Our findings indicate that HD-tDCS alters brain activity and affects the resting brain network. Studies have investigated the effects of repeated tDCS combined with cognitive training, suggesting that tDCS effects may not always be additive in memory rehabilitation programs [82]. In summary, the present study supports previous findings [83, 84] indicating the impact of tDCS on cognition. Delivering repeated HD-tDCS to the L-DLPFC of individuals with MCI provided insight into resting brain network changes. Lu H et al. found that repeated anodal HD-tDCS provides a positive benefit on executive control and psychomotor efficiency and has obvious accumulative effect after nine or more times intervention compared to sham HD-tDCS [85]. This information may prove useful for future clinical applications of HD-tDCS to improve cognitive function.

No significant correlations were found between ReHo or fALFF values and MMSE or MoCA scores among the two groups. The reason for this could be as follows: First, the duration of HD-tDCS is not sufficient. A recent study suggested that repeated anodal-tDCS (10 sessions) induces cumulative effects on the cognitive performance of patients with brain injury, and significant superiority in the anodal-tDCS group appeared after two weeks or one month after the end of the stimulation, which may reflect the difference seen in this study [86]. Second, the current intensity is an influencing factor. A study with 1 mA of central electrode stimulation at the right DLPFC reported significant findings [87] while Bayesian statistics show a lack of change in excitability following bi-hemispheric HD-TDCS with 1.5mA current over the primary somatosensory cortices [88]. Sedgmond J et al found that no evidence that HD-tDCS with a 1.5 mA current in the prefrontal influences cue-induced food craving [89]. Breitling-Ziegler C et al found that distinct effects of tDCS with different current intensities demonstrating the importance of a deeper understanding on the impact of stimulation parameters and repeated tDCS applications to develop effective tDCS-based therapy approaches [90]. Low stimulation intensity might be a possible reason for the lack of effects seen. Future research should consider this. Third, the evaluation scale may not have been sufficiently accurate, such that slight changes may not have been discovered in time.

### Limitations and future considerations

Our study has several limitations. First, the sample size was small, and a small sample size can lead to statistical errors. More studies with a larger sample size should be undertaken in the future. Second, our functional MRI data were not evaluated with biochemical indicators. If combined with biochemical indicators, the impact of HD-tDCS on brain activity may be more clearly revealed. Third, during HD-tDCS, the task-state fMRI data could not be collected and analyzed. We only analyzed the resting-state functional MRI data. If this data were combined with the fMRI changes in the task state, we could better understand how HD-tDCS affects the internal activities of the brain. Fourth, the cognitive assessment scale was relatively simple. It would be easier for us to observe the effects of HD-tDCS on cognitive behavior if we used a more accurate scale for assessing cognitive function.

## Conclusions

In summary, the present study demonstrated that repeated HD-tDCS modulates functional brain activity not only in the area close to the stimulation site but also in more remote areas. In this study, we used fALFF and ReHo to measure the brain’s functional activity and found alterations in related brain regions. More specifically, the altered fALFF and ReHo indices were located in the key nodes of the default mode network and the right frontal-parietal network. This finding may indicate that HD-tDCS lowers the default mode network and activates the right frontal-parietal network. These regions help participants selectively respond to external stimulation and perform tasks they are interested in. These findings add to our understanding of the mechanism of action of HD-tDCS, specifically in terms of HD-tDCS affecting resting brain network indicators. Interestingly, HD-tDCS increased the ReHo values of multiple brain regions, which may be related to the underlying mechanism of its clinical effects, and may be related to a potential compensation mechanism involving mobilizing more regions to complete a function after the function declines.
